# The Nature of Available Choices Affects the Intake and Meal Patterns of Rats Offered a Palatable Cafeteria-Style Diet

**DOI:** 10.3390/nu15245093

**Published:** 2023-12-13

**Authors:** Carolina R. Cawthon, Alan C. Spector

**Affiliations:** Department of Psychology and Program in Neuroscience, Florida State University, Tallahassee, FL 32304, USA; cawthon@psy.fsu.edu

**Keywords:** cafeteria diet, eating patterns, macronutrient intake, food choice

## Abstract

Humans choose which foods they will eat from multiple options. The use of cafeteria-style diets with rodent models has increased our understanding of how a multichoice food environment affects eating and health. However, the wide variances in energy density, texture, and the content of micronutrients, fiber, and protein can be interpretatively problematic when human foodstuffs are used to create rodent cafeteria diets. We minimized these differences with a custom rodent cafeteria diet (ROD) that varied similarly to a previously used human-foods cafeteria diet (HUM) in fat and sugar content. Here, we used our custom Five-Item Food Choice Monitor to compare the intake and meal patterns of rats offered ROD and HUM in a crossover design. Compared with chow, rats consumed more calories, sugar, and fat and less protein and carbohydrate while on either of the choice diets (*p* < 0.05). While energy intake was similar between HUM and ROD, there were differences in the responses. Rats consumed more of the low-fat, low-sugar choice on the ROD compared with the nutritionally similar choice on the HUM leading to differences in fat and carbohydrate intake between the diets (*p* < 0.05). The stability of macronutrient intake while on either choice diet suggests macronutrient intake is determined by the available foods and is strongly regulated. Therefore, interpretative consideration must be given to the nature of food choices in the context of available options when interpreting cafeteria-diet intake.

## 1. Introduction

Animal models consistently provide a well-controlled and valuable way to interrogate ingestive behavior. Many clinical disorders include some relationship to nutrient intake and better modeling of the human-food environment can increase translatability of preclinical results. In particular, humans select what foods to eat from multiple options, and the provision of choice is one way to better emulate human eating conditions in rodent models. The use of cafeteria-style diets with rodent models has increased our understanding of how a multichoice food environment affects eating and health. Humans eat more when variety is increased, e.g., [1,2], and this behavior is replicated in rodents offered multichoice diets, e.g., [2,3,4,5]. The availability of food choices has also been shown to affect or interact with memory, e.g., [6,7], anxiety and depression, e.g., [8], and stress responses e.g., [9] in addition to meal patterns e.g., [10], and subsequent food preferences, e.g., [10,11,12]. Fully understanding the effects of food selection in an environment with multiple choices is fostered by the use of cafeteria-style diets in preclinical models. However, consideration of how various properties of the choices interact to drive selection is also interpretatively important, and a better understanding of these factors can improve the design of interventions for obesity and other conditions. For example, despite recent significant advances in treatment that are reviewed in references [13,14,15], obesity can be treated more effectively when lifestyle factors such as food choice are addressed.

In some cases, including earlier work by our lab, human foodstuffs have been used to provide diet choices in rat models of ingestive behavior, for example, in [3,6,10,16,17,18,19,20]. However, along with the desired variety in nutritional characteristics, human foods come with wide variability in ingredients and texture. Depending on the research question and specific manipulation, having many differences in a variety of food characteristics introduces interpretive challenges. For example, based on the diets used historically in our own laboratory, if a manipulation led to an increased intake of low-fat vanilla yogurt and decreased intake of chow, would this be the result of increased avidity for sugar, for foods with higher water content, or for the fatty-acid profile of the yogurt compared with that of the chow? This is but one example provided for illustration, but it does make clear that determining changes in the regulation of macronutrient intake can be difficult if food choices vary too much in other ways. To our knowledge, there is no research investigating the response to a cafeteria diet where variation in food characteristics is limited to key factors of interest. Additionally, research comparing the responses of rats to more than one cafeteria-style diet is lacking; thus, it is not known if rats choose from among the available choices solely on the basis of macronutrient needs or if other characteristics of the choices are important.

Aiming to minimize all but the key macronutrient variations, Research Diets (New Brunswick, NJ, USA) helped us devise custom rodent diets that would minimize or eliminate differences in all characteristics except sugar and fat content, which would vary similarly to the human-food-based cafeteria diet we have used in the past [16,17]. Here, we presented rats with either an array of the human foods used previously [3,16,17] or an array of the custom rodent foods and compared the food choice, relative macronutrient intake, and meal patterns associated with the two diet arrays. These diet arrays (rodent or human) were each given to male and female rats for 8 days in a crossover design using a specially designed Five-item Food Choice Monitor (FCM) [3]. We aimed to determine if the intake and meal patterns of these cafeteria diets were similar when compared both to standard chow and to each other and to assess the utility of the new custom rodent choice diet to serve as a palatable cafeteria-style diet.

## 2. Materials and Methods

### 2.1. Subjects

Subjects in the diet crossover feeding monitoring study were 16 Sprague–Dawley rats (8 male and 8 female), approximately 7 weeks of age on arrival. The day after arrival, the female rats weighed 141–189 g and the male rats weighed 183–262 g. A second set of 6 male Sprague–Dawley rats, 11 weeks old (weighing 333–404 g) and naïve to the diets, was used in 2-day 2-jar preference tests of nutritionally similar foods.

The rats were single-housed in regular or modified polycarbonate tub cages in a light, temperature, and humidity-controlled vivarium with lights on from 07:00 to 19:00. The rats had ad libitum access to food and water while in standard cages, and a stainless-steel toy was provided for environmental enrichment. While housed in the FCM, food and water were available for 22 h/day, as described in Section 2.2, and a stainless-steel nesting area was provided for enrichment. During all but the 2-day 2-jar preference tests, standard rodent chow was always one of the available food choices and at least one choice provided appropriate protein, vitamins, and minerals for the maintenance of rat health.

All procedures were approved by the Florida State University Animal Care and Use Committee.

### 2.2. Apparatus

**Figure 1:**
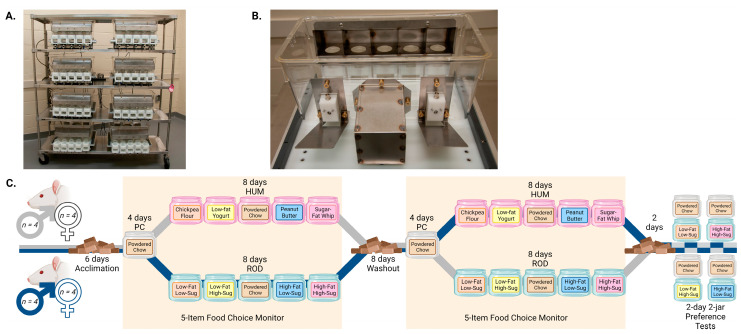
The 5-Item Food Choice Monitor (FCM) and Experimental Timeline. (**A**). Rack of 8 monitoring cages that are controlled by a single computer, not shown. (**B**). View of a single food monitoring cage. Each of the 5 circular cutouts in the compartmentalized food access hood provides access for the rat to eat from one of 5 food jars that is held on a load beam beneath the cutout. Opposite the food access hood are 2 lick blocks on either side of the stainless-steel nest for the rat. The FCM is fully described in Section 2.2. (**C**). Experimental Timeline. Periods when the rats were in the FCM are shaded yellow; periods with white backgrounds were conducted in standard cages. The foods offered are more fully described in Section 2.3 and Table 1, the experimental design is further explained in Section 2.4, and the method used to conduct the 2-day 2-jar preference tests is described in Section 2.5. PC = powdered chow; HUM = human-foods choice-diet array; ROD = custom rodent choice-diet array. The experimental timeline was designed in BioRender.com.

At specified times, the rats were placed into a custom-designed, recently validated FCM (FCM, Figure 1; thoroughly described in [3]). Briefly, the FCM provides continuous recording of changes in weight of up to 5 food choices and licks from 2 fluid bottles, allowing a detailed analysis of what, when, how much, and at what rate rats eat and drink. The system uses standard polycarbonate rat cages with modifications to allow access to five foods via a stainless-steel hood with 5 separated compartments that enable the rats to eat while preventing a mixing of foods. Food-jar weight changes are measured 10 times per second and collected in 10 s bins. Opposite the feeding hood are two lick blocks that continuously record licking to two fluid bottles. Intake measurements occurred for 22 h daily, with the remaining 2 h per day for animal care and FCM maintenance. During this study, the 2 h maintenance period occurred from approximately 11:30 to 13:30 each day (beginning 4.5 h into the light period). To prevent position bias, the locations of food jars and fluid bottles were rotated daily. A nest area, between the water bottles, provides environmental enrichment for the animal. In addition to FCM recordings, food jars and water bottles were manually weighed before placement and after removal to provide external validation of the intake measures.

### 2.3. Foods

Deionized water and standard laboratory chow were continuously available to the rats unless noted otherwise. Specifically, the rats could not access food and water during daily maintenance when in the FCM, and the rats used for the 2-jar tests of nutritionally similar foods did not receive chow during comparison testing. While in the feeding monitoring system, rats had access to five different foods (i.e., powdered rodent chow (PC, LabDiet 5001) plus 4 additional choices). One set of foods consisted of 4 custom rodent diets (ROD; Research Diets, New Brunswick, NJ, USA) that were either high (H) or low (L) in fat (F) and/or sugar (S) (i.e., LFLS, LFHS, HFLS, and HFHS). The second group of foods consisted of human foodstuffs (HUM) used previously by the laboratory (e.g., [3,16]). The HUM diet choices were chickpea flour (CF, low-fat, low-sugar, Bob’s Red Mill), low-fat vanilla yogurt (Y, low-fat, high-sugar, Winn-Dixie), creamy peanut butter (PB, low-sugar, high-fat, Jif), and a custom sugar–fat whip (SFW, high-fat, high sugar) that resembles frosting or sandwich-cookie filling. The human foods were originally selected to represent prototypical fat and sugar contents while being comparable in protein content and to meet practical considerations, such as resistance to removal from food jars for hoarding rather than eating and minimal spillage and spoilage. We asked Research Diets to devise custom rodent diets having standardized protein, fiber, electrolyte, vitamin, and mineral contents but with variability in sugar and fat similar to the human-food-based cafeteria diet the lab has used in the past [16,17]. To further increase similarity, all sugar in the ROD options was from sucrose, nonsugar carbohydrate was from cornstarch, and the fat was consistently 10% from soybean oil and 90% from lard, producing proportionally identical fatty-acid profiles. Nutritional details for each food choice are given in Table 1.

### 2.4. Experimental Design

The rats were allowed a 6-day period of acclimation to our facility. Pelleted-chow intake and body weight were measured daily during acclimation. After acclimation, the rats were placed into our FCM. After 4 days in the apparatus, during which the rats received powdered rodent chow only, one half of the rats (groups matched by weight and sex) were given the ROD diet while the other half received the HUM diet. After 8 days on the choice diets, the rats were returned to standard cages and pelleted chow for an 8-day washout period. The washout was included so that the rats would start the second FCM testing period in a physiological state more like when they began the first cafeteria phase rather than in a state of increased weight gain. During washout, pelleted-chow intake and body weight were measured daily. After washout, the rats were returned to the FCM and given 4 days of powdered chow before being offered the opposite choice diet from their first FCM session. We used a crossover design to account for differences caused by which diet was offered first. At the conclusion of food-choice monitoring, the rats were returned to regular cages for two days and then divided by sex and order of diet exposure for a series of 48 h 2-jar tests to assess preference for the custom rodent diets compared to powdered chow, since they were not tested previously. Preference for the human-food choices compared to chow has been reported [3]. The experimental timeline is depicted in Figure 1C.

### 2.5. Two-Jar Tests

To assess whether rats found the custom ROD choices more palatable than chow, we offered each food with only powdered rodent chow as an alternative after the conclusion of food-choice monitoring. Two-jar tests were conducted in home cages, with foods placed in jars wired to the side of the cage to prevent tipping. Each of the 4 rodent diets was offered against powdered chow for 2 days, and each pair was tested by 4 male and 4 female rats that had participated in the crossover food-choice monitoring experiment. Two jars, half filled with either PC or one of the ROD foods, were weighed, and then, one was placed in one front cage corner and the other was placed in the opposite front corner. After 24 h, the rats and jars were weighed, then the jars were refilled, weighed, and replaced in the cage with the positions switched. At the end of the second 24 h, the jars were weighed and, except for after the last choice, a new ROD choice was tested vs. powdered chow.

After an analysis of intake by the rats during the diet crossover experiment showed that the rats consumed differing proportions of the low-sugar food choices between the diets, we conducted a second 2-day 2-jar test using 6 naïve male Sprague–Dawley rats. The protocol was as described above, except the rats began the testing with 2 jars of powdered chow; then, instead of one choice being powdered chow, the rats had the nutritionally similar foods from each diet array as their two choices (i.e., foods found in the same row of Table 1: CF vs. LFLS, Y vs. LFHS, PB vs. HFLS, and SFW vs. HFHS).

### 2.6. FCM Output and Statistical Analysis

Raw output from the FCM was analyzed using specialized software that allows the investigator to customize meal parameters, including minimum meal size (defined as 1 kcal) and the meal pause termination criterion, which was defined as 900 s without intake. The 900 s intermeal interval has been used in other studies [21,22,23,24,25] and is consistent with a series of pause interval analyses conducted in our lab using human foodstuffs [3].

Statistical testing was conducted using Systat 13 (Inpixion, Palo Alto, CA, USA). Body weights were compared within each sex using a repeated-measures ANOVA. Due to variability, all other data were analyzed using Friedman’s Test, and Bonferroni-corrected *p*-values are reported. Within each sex, means for each 8-day choice-diet testing phase were compared to PC means and to each other, the first and last day of each diet phase was compared to PC, the first day of a diet phase was compared to the last day of the diet phase, and the first day of HUM was compared to the first day of ROD. For each measure, we used the Mann–Whitney U Test to compare males to females.

## 3. Results

### 3.1. Body Weight

**Figure 2:**
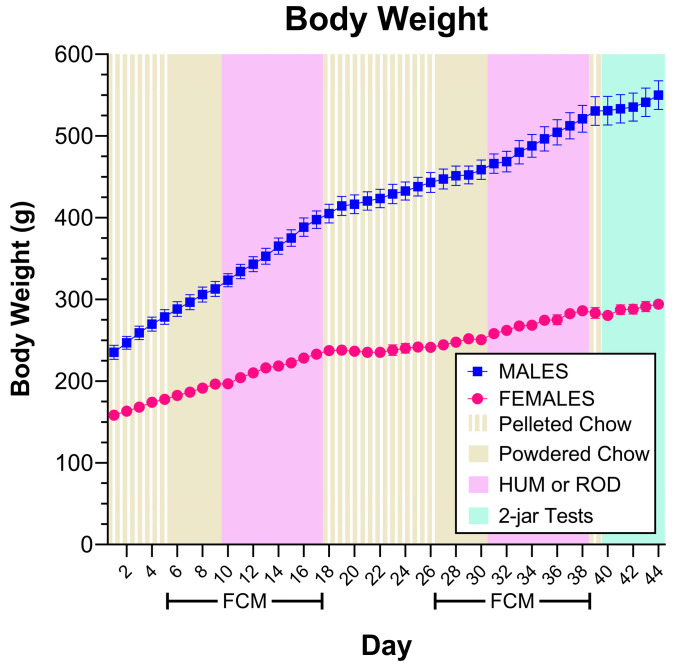
Rats of the same sex remained similar in body weight. Body weight for each sex group shown as MEAN ± SE. Males are shown with filled symbols and the different experimental phases are shaded with colors identified in the legend. FCM = food-choice monitor; HUM = human-foods choice diet; ROD = custom rodent choice diet. Comparing rats of the same sex based on the order they received the cafeteria diets using repeated measures ANOVA does not find differences (Males, effect of order F (1, 6) = 0.0873, *p* = 0.7776, effect of day, F (43, 258) = 436.9651, *p* < 0.0001, order X-day interaction F (43, 258) = 3.2340, *p* = 0.0721. Females, effect of order F (1, 6) = 0.0248, *p* = 0.8800, effect of day, F (43, 258) = 167.0638, *p* < 0.0001, order X-day interaction F (43, 258) = 0.5066, *p* = 0.7769).

Rats of the same sex remained similar in body weight throughout the study (Figure 2). The lack of differences in body weight across diets suggests that, overall, rats responded similarly to the HUM and ROD diets.

### 3.2. Energy Intake

**Figure 3:**
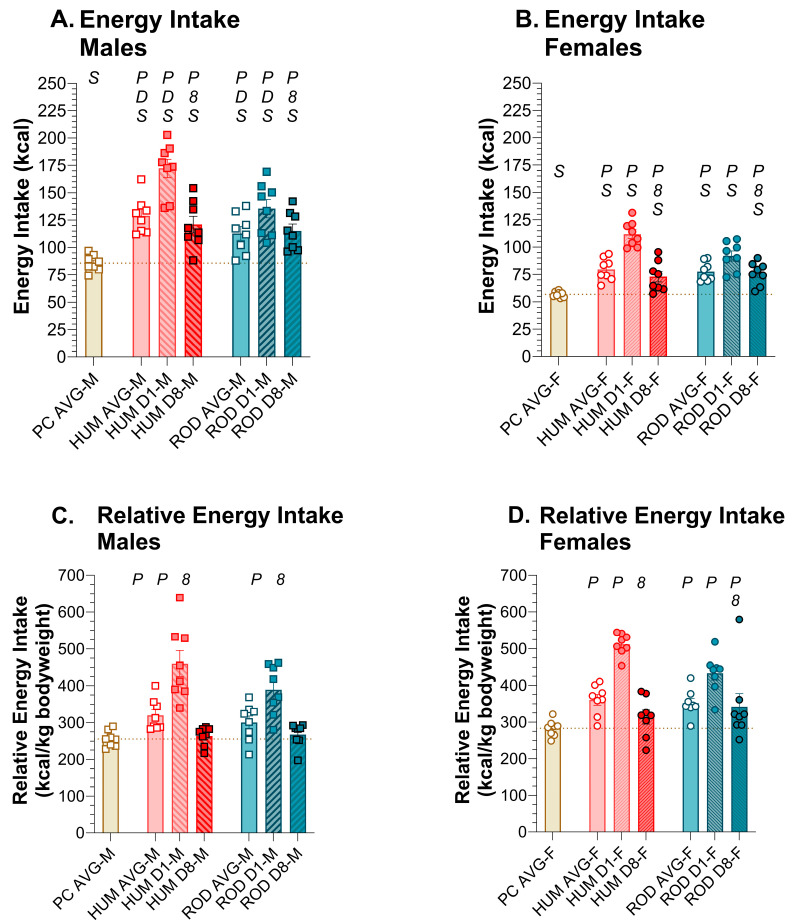
Energy intake increased with choice diets. Average energy intake for powdered chow (PC, tan bar), the human-foods diet array (HUM, orange bars), and the custom rodent diet array (ROD, teal bars) and intake on the first (D1) and last (D8) day of each choice diet shown as MEAN ± SE. Individual animals are represented by squares (males) or circles (females). The tan horizontal dashed line represents the mean for PC. The letters/numbers above the bars denote Bonferroni-corrected statistical differences: *P* = ROD/HUM vs. PC average; *D* = ROD/HUM vs. each other; *8* = day 8 vs. day 1; *S* = males vs. females. Statistical details are in Table 2 and Table 3. (**A**). Energy intake of male rats. (**B**). Energy intake of female rats. (**C**). Relative energy intake of male rats. (**D**). Relative energy intake of female rats.

Male and female rats consumed more calories while on the choice diets than they did on powdered chow alone (Figure 3, Table 2 and Table 3). Since the male rats were larger than the female rats, their absolute energy intake was greater but if we assess energy intake relative to body weight, we do not find sex differences or, within each sex, differences between the choice diets. These findings support the conclusion that both the HUM and ROD diets are suitable for service as palatable choice diets.

Whether we look at absolute or relative energy intake, there was a dramatic increase in energy intake on the first day of choice-diet exposure in both sexes, with energy intake declining over the duration of the 8-day exposure. By Day 8, the relative energy intake of HUM by males and females returned to levels similar to PC alone. On the ROD diet, relative kcal intake of males returned to PC intake levels by Day 8, but in females, it remained higher than PC alone.

### 3.3. Water Intake

**Figure 4:**
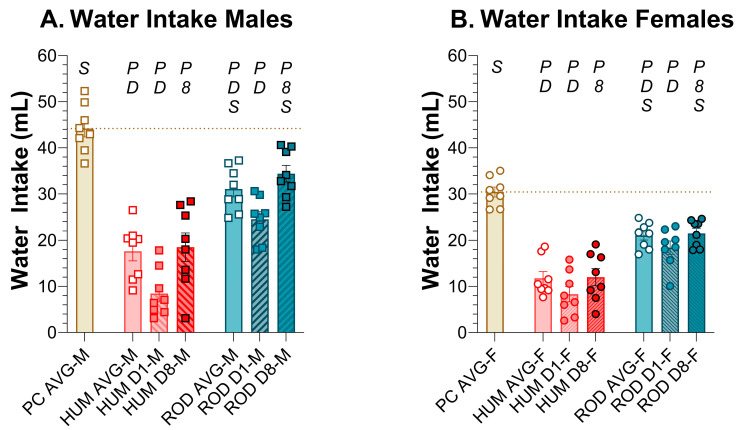
Water intake decreased on choice diets. Average water intake for the powdered chow (PC, tan bar), the human-foods diet array (HUM, orange bars), and the custom rodent diet array (ROD, teal bars) phases and intake on the first (D1) and last (D8) day of each choice diet shown as MEAN ± SE. Individual animals are represented by squares (males) or circles (females). The tan horizontal dashed line represents the mean for PC. The letters/numbers above the bars denote Bonferroni-corrected statistical differences: *P* = ROD/HUM vs. PC average; *D* = ROD/HUM vs. each other; *8* = day 8 vs. day 1; *S* = males vs. females. Statistical details are in Table 2 and Table 3. (**A**). Water intake of male rats. (**B**). Water intake of female rats.

The male and female rats consumed less water on the HUM and ROD diets than when on powdered chow alone. The rats of both sexes drank less on the HUM diet than the ROD diet (Figure 4, Table 2 and Table 3), likely due to its inclusion of low-fat vanilla yogurt, which has a higher water content relative to the other choices in either diet array.

### 3.4. Intake of Comparable Foods: ROD and HUM

**Figure 5:**
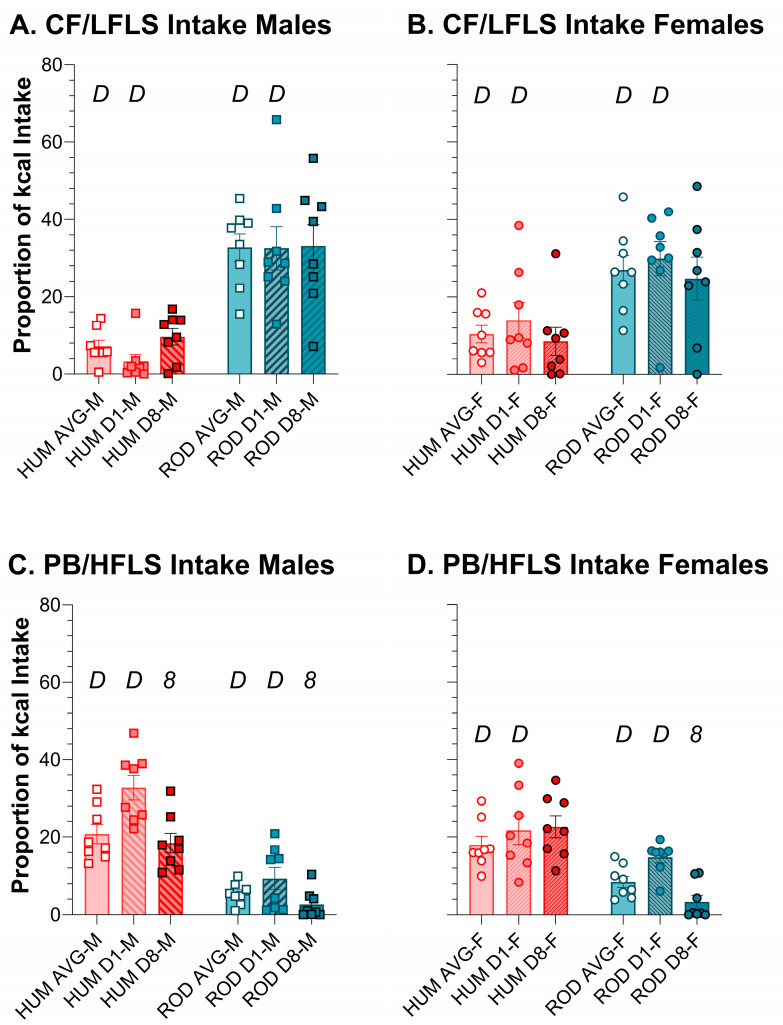
Average intake of the low-sugar choices differed between the HUM (orange bars) and ROD diets (teal bars) for males (squares) and females (circles). Average intake and intake on Day 1 and Day 8 of each choice diet shown as a proportion of total kcal (MEAN ± SE). Nutritionally similar food choices between the HUM and ROD diets are shown side by side (nutritional details can be found in Table 1). ROD choices were high (H) or low (L) in fat (F) and/or sugar (S). CF = chickpea flour; PB = creamy peanut butter. Bonferroni-corrected statistical differences: *D* = ROD/HUM vs. each other; *8* = day 8 vs. day 1. Statistical details are in Table 4 and Table 5. CF/LFLS intake of (**A**) males and (**B**) females. PB/HFLS intake of (**C**) males and (**D**) females.

**Figure 6:**
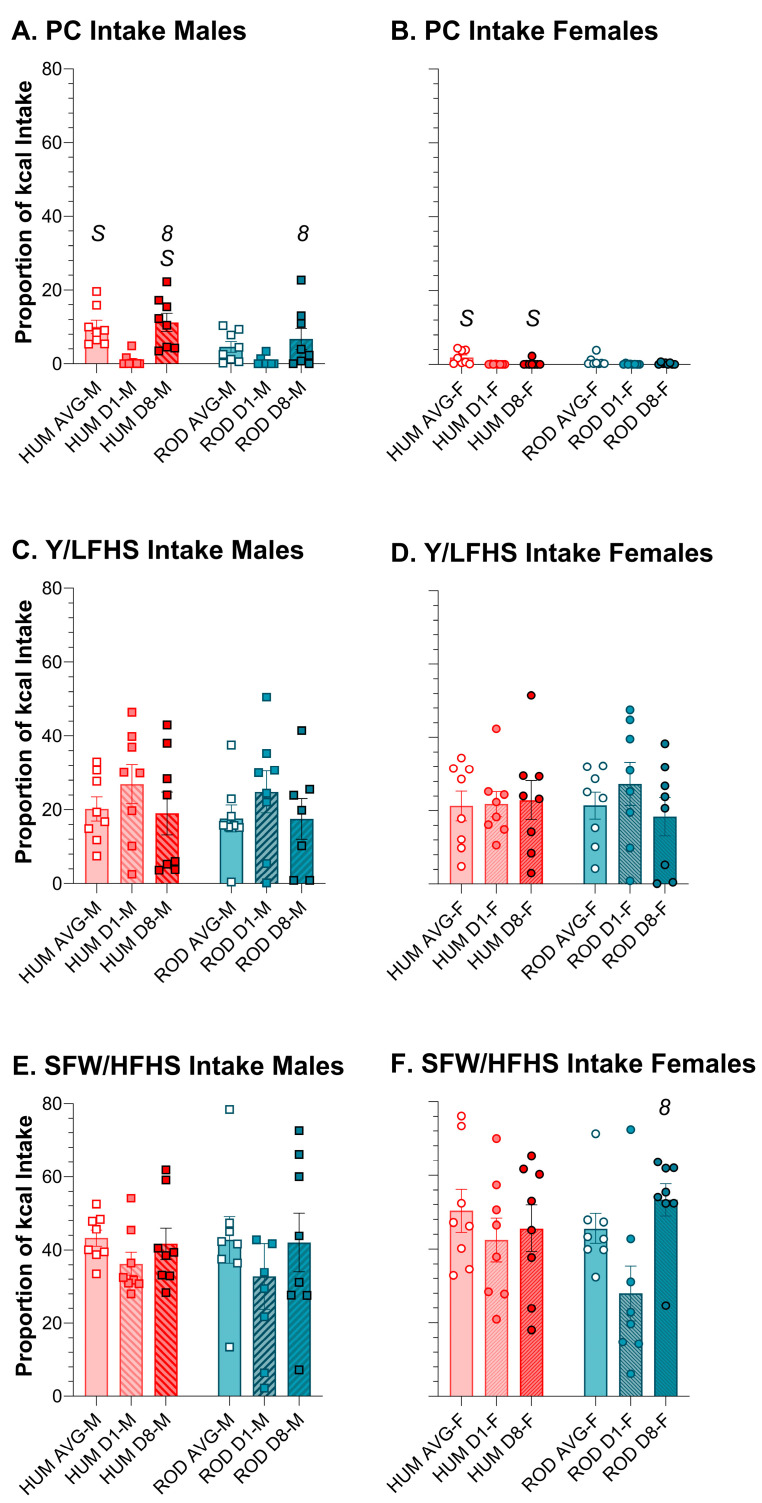
Average intake of the high-sugar choices and powdered chow was generally similar between the ROD (teal bars) and HUM (orange bars) diets for males (squares) and females (circles). Average intake and intake on Day 1 and Day 8 of each choice diet shown as a proportion of total kcal (MEAN ± SE). Nutritionally similar food choices between the HUM and ROD diets are shown side by side (nutritional details can be found in Table 1). ROD choices were high (H) or low (L) in fat (F) and/or sugar (S). PC = Powdered Chow; Y = Low-fat vanilla yogurt; SFW = Sugar–fat whip. Bonferroni-corrected statistical differences: *8* = day 8 vs. day 1; *S* = males vs. females. Statistical details are in Table 4 and Table 5. PC intake of (**A**) males and (**B**) females. Y/LFHS intake of (**C**) males and (**D**) females. SF/HFHS intake of (**E**) males and (**F**) females.

Using relative intake as a proxy for preference, on ROD the order of food preference for males and females was HFHS > LFLS > LFHS > HFLS > PC (Figure 5 and Figure 6). On HUM, the order of preference for male rats was SFW > PB ≥ Y > PC ≥ CF. Although female rats also most preferred SFW from the HUM array, their order of preference differed slightly from the males: SFW > Y ≥ PB > CF > PC. Given the similarity in sugar and fat content between the choices in the diet arrays, it appears that what the foods are matters (Figure 5 and Figure 6, Table 4 and Table 5). Over time, the intake of individual foods tended to be stable with only a few exceptions. Male rats tended to increase intake of PC between Day 1 and Day 8 on both diet arrays, and this was consistently concurrent with reducing intake of the choice that was high in fat and low in sugar (Figure 5 and Figure 6, Table 4 and Table 5). Females also reduced intake of the HFLS choice on ROD, but, rather than PC like the males, they increased HFHS intake instead (Figure 5 and Figure 6, Table 4 and Table 5).

### 3.5. Macronutrients and Sugar Intake: ROD, HUM, and PC

**Figure 7:**
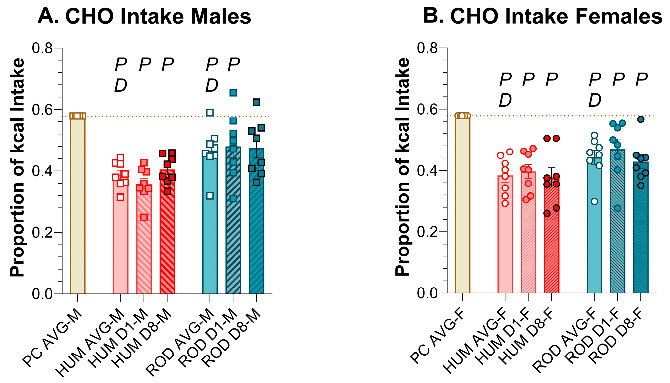

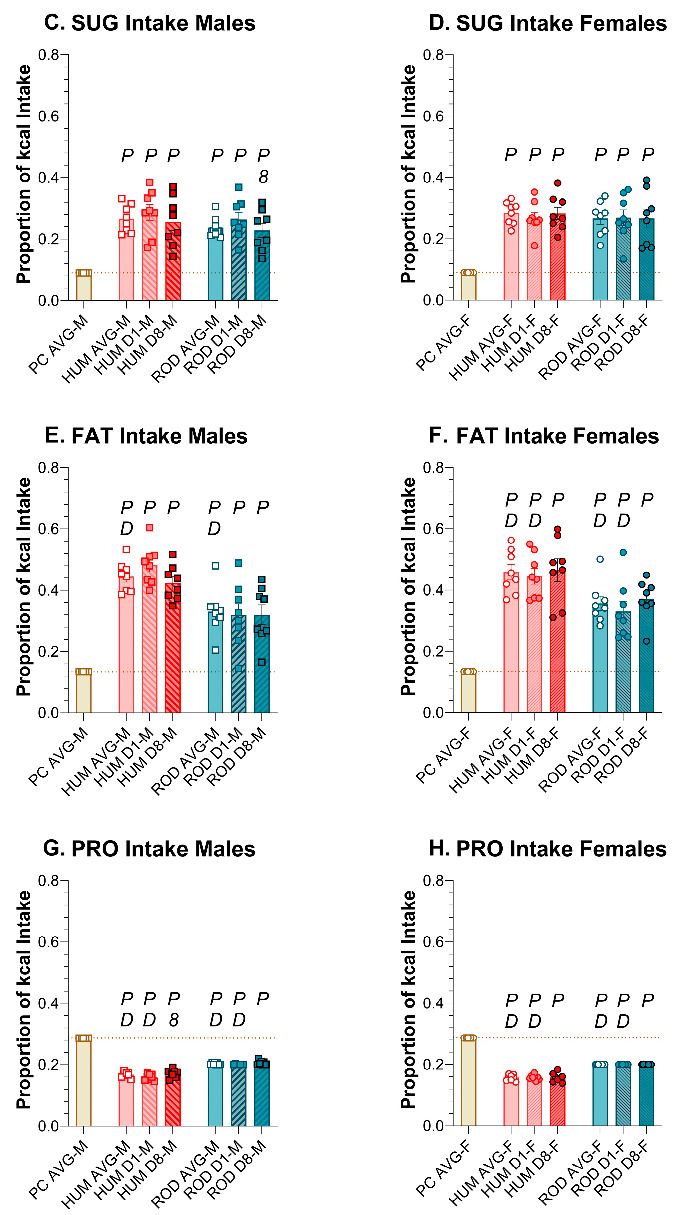
Macronutrient intake on the ROD (teal bars) and HUM (orange bars) diets differed from powdered chow (PC, tan bar) and from each other. Graphs show MEAN ± SE intake of total carbohydrate (CHO), sugar (SUG), fat, and protein (PRO) as averages for each diet phase and on Day 1 and Day 8 of each choice diet. Individual animals are represented by squares (males) or circles (females). The tan dashed horizontal line represents the mean for PC. The letters/numbers above the bars denote Bonferroni-corrected statistical differences: *P* = ROD/HUM vs. PC average; *D* = ROD/HUM vs. each other; *8* = day 8 vs. day 1. Statistical details are in Table 6 and Table 7. (**A**) CHO intake of male rats. (**B**) CHO intake of female rats. (**C**) SUG intake of male rats. (**D**) SUG intake of female rats. (**E**). Fat intake of male rats. (**F**). Fat intake of female rats. (**G**). PRO intake of male rats. (**H**). PRO intake of female rats.

Compared to PC alone, male and female rats consumed less total CHO and PRO and more SUG and FAT on both the HUM and ROD diets (Figure 7, Table 6 and Table 7). Except for a slight decrease in sugar intake on the ROD diet and a slight increase in protein intake on the HUM diet across days in males, the macronutrient intake did not vary between Day 1 and Day 8 on either choice diet. In males and females, the sugar intake did not differ between the HUM diet and the ROD diet (Figure 7C,D, Table 6 and Table 7).

A comparison of the choice diets to each other shows that total CHO intake was greater on the ROD diet than the HUM diet for both sexes (Figure 7A,B, Table 6 and Table 7). PRO intake was also greater on ROD compared to HUM, while FAT intake was less on ROD than HUM. Thus, the macronutrient intake profile depended on which set of diets were available.

### 3.6. Meal Characteristics

#### 3.6.1. Number of Meals

**Figure 8:**
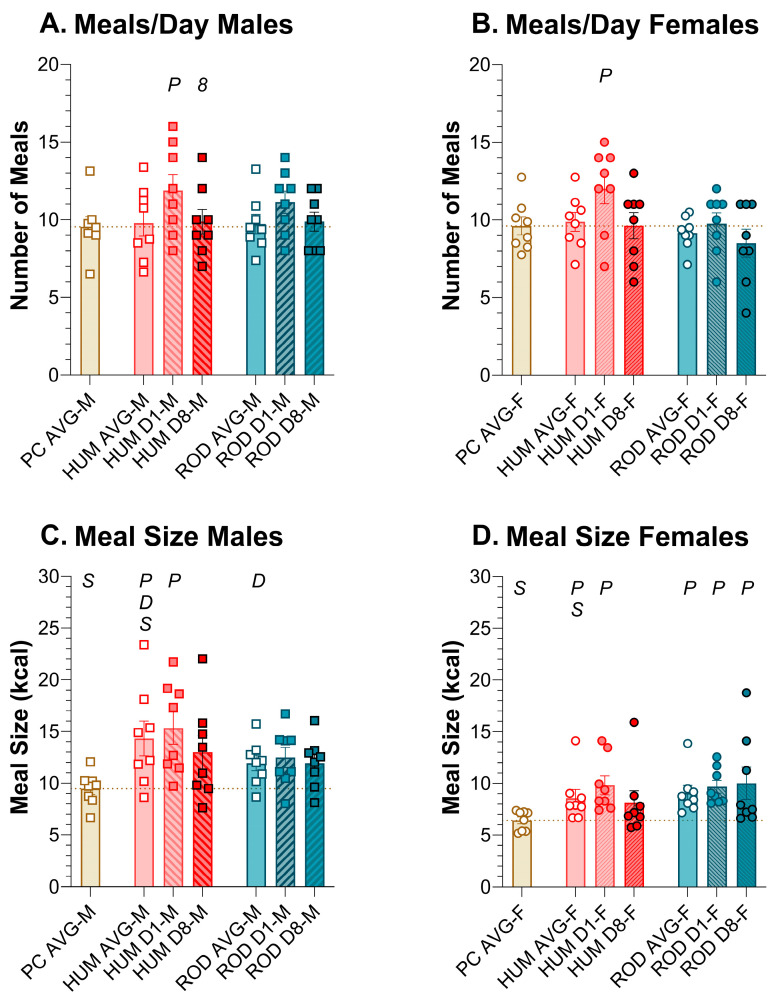
Effects on meal number and meal size were responsible for Day 1 increases in intake. Graphs show meal number and meal size as MEAN ± SE for the average during each diet phase (powdered chow (PC, tan bar), the human-foods diet array (HUM, orange bars), and the custom rodent diet array (ROD, teal bars) and on Day 1 and Day 8 of each choice diet. Individual animals are represented by squares (males) or circles (females). The tan dashed horizontal line represents the mean for PC. The letters/numbers above the bars denote Bonferroni-corrected statistical differences: *P* = ROD/HUM vs. PC average; *D* = ROD/HUM vs. each other; *8* = day 8 vs. day 1; *S* = males vs. females. Statistical details are in Table 8 and Table 9. Number of meals per day in (**A**) male rats and (**B**) female rats. Meal size of (**C**) male rats and (**D**) female rats.

In males and females, the average number of meals per day was consistent across PC, HUM, and ROD (Figure 8A,B, Table 8 and Table 9). However, on HUM Day 1, rats of both sexes consumed more meals than on PC. In males, there was a trend (*p* = 0.043) toward an increase in meal number on ROD Day 1 that did not survive Bonferroni correction.

#### 3.6.2. Meal Size

In males, average meal size (kcal) was greater on HUM than PC or ROD and did not differ between PC and ROD (Figure 8C,D, Table 8 and Table 9). In females, the average meal size (kcal) for PC was lower than either HUM or ROD, which, in turn, did not differ. By HUM Day 8, meal size in males and females was not statistically different from PC alone or HUM Day 1. Unlike males, meal size in females remained above that of PC alone while on the ROD diet.

#### 3.6.3. Meal Duration

**Figure 9:**
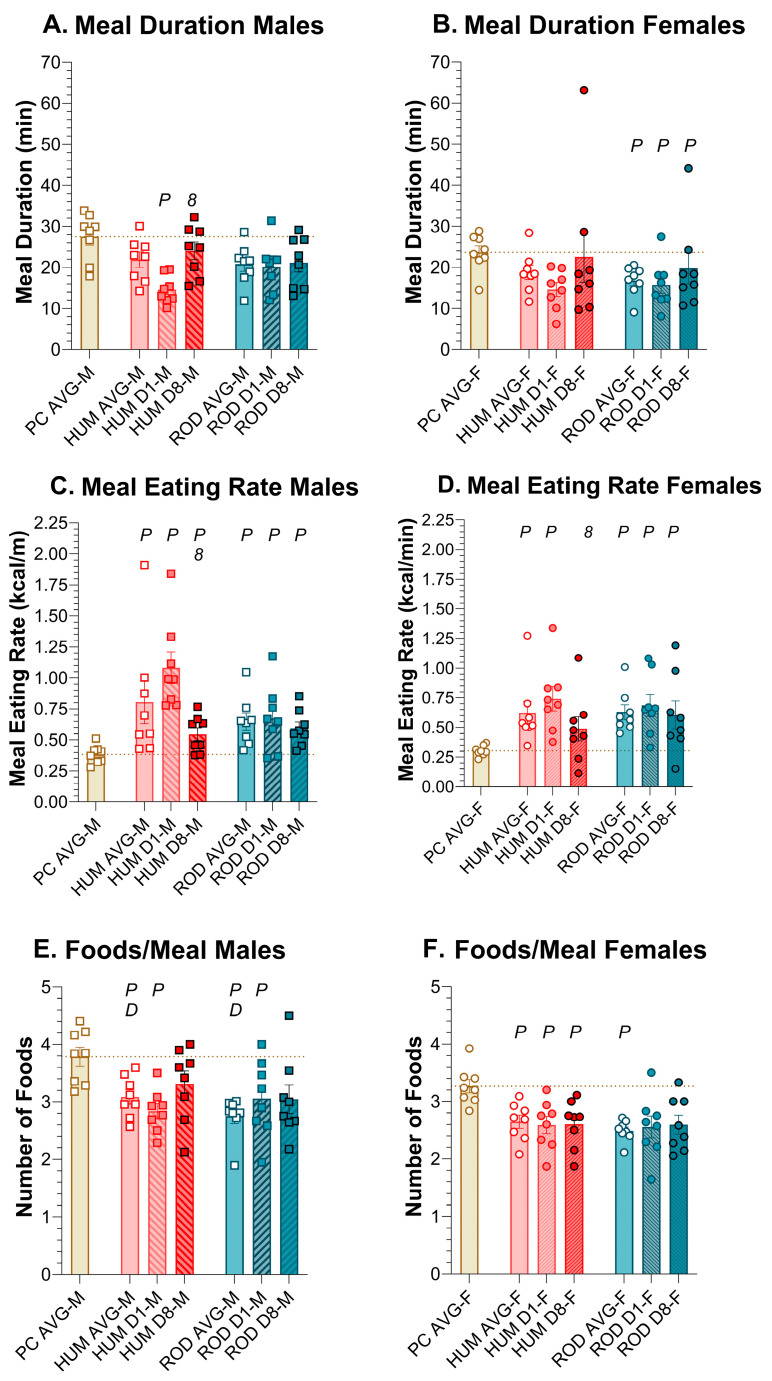
Meal duration, eating rate, and foods per meal. Graphs show meal duration, eating rate, and number of foods/meals as MEAN ± SE for the average during each diet phase (powdered chow (PC, tan bar), the human-foods diet array (HUM, orange bars), and the custom rodent diet array (ROD, teal bars) and on Day 1 and Day 8 of each choice diet. Individual animals are represented by squares (males) or circles (females). The tan dashed horizontal line represents the mean for PC. The letters/numbers above the bars denote Bonferroni-corrected statistical differences: *P* = ROD/HUM vs. PC average; *D* = ROD/HUM vs. each other; *8* = day 8 vs. day 1. Statistical details are in Table 9 and Table 10. Meal duration of (**A**) male and (**B**) female rats. Meal eating rate of (**C**) male and (**D**) female rats. Number of foods/meal of (**E**) Male and (**F**) Female rats. For panels (**E**,**F**), the tan bar and tan dashed line represent the mean number of jars of PC engaged.

The average meal duration did not vary between PC, HUM, and ROD in males, but on HUM Day 1, in males, the meal duration was significantly shorter than on PC alone or HUM Day 8 (Figure 9A,B, Table 9 and Table 10). In females, the HUM meal duration was consistently similar to PC, but the ROD meal duration was consistently shorter than for PC.

#### 3.6.4. Meal Rate

In males and females, the average meal ingestion rate was increased on HUM and ROD compared to PC alone, and there was no difference between HUM and ROD (Figure 9C,D, Table 9 and Table 10). In both sexes, on HUM, the ingestion rate declined between Day 1 and Day 8, becoming similar to PC in females. On the ROD diet, the ingestion rate persisted above PC levels in males and females.

#### 3.6.5. Number of Foods Per Meal

When rats were on PC alone, they were given five jars containing PC, and the FCM reported how many jars they ate from during a meal thus reporting the number of jars a rat would eat from when the available food was the same. In males and females, on average, the provision of either choice diet resulted in rats choosing fewer jars (i.e., foods) compared to PC alone. And, on the HUM diet, females chose fewer foods than males (Figure 9E,F, Table 9 and Table 10). In general, however, all rats on average sampled from close to three jars per meal when on the ROD and HUM diets.

### 3.7. Two-Jar Tests

#### 3.7.1. ROD v PC

**Figure 10:**
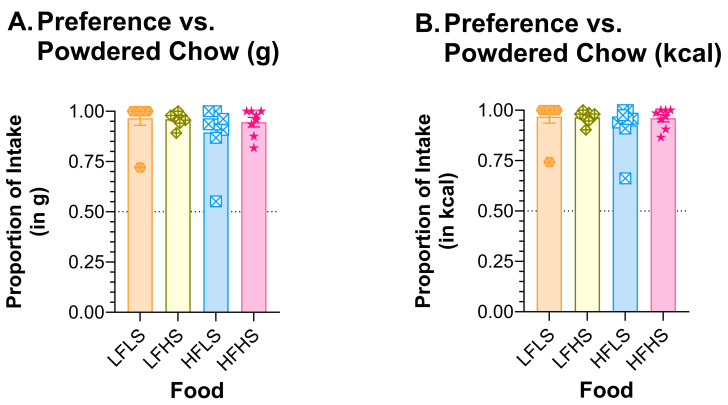
Rats preferred all ROD choices to PC. Graph shows MEAN ± SE proportion of (**A**) mass or (**B**) kcal eaten from each ROD choice when offered with only PC as the other food choice. The dashed line at 0.50 shows the point of equal preference; values above this line indicate greater preference. The bar colors match the food jar labels in Figure 1C. Individual rat preferences are shown as symbols in each bar. ROD choices were high (H) or low (L) in fat (F) and/or sugar (S).

All ROD food choices were preferred to PC by male and female rats (Figure 10). If preference is calculated as a function of grams consumed, the preference for all ROD foods was ≥0.895. If calculated as a function of kcal consumed, the preference for ROD foods was ≥0.925.

#### 3.7.2. ROD v HUM

**Figure 11:**
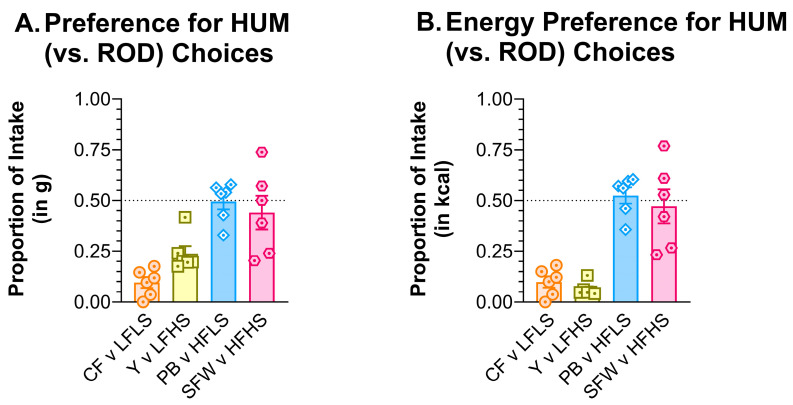
Rats preferred the ROD choices that were low in fat over the low-fat HUM foods. Graph shows MEAN ± SE proportion of (**A**) grams or (**B**) the proportion of total kcal eaten from each HUM food choice when offered with only the nutritionally similar ROD option as the other food choice. The dashed line at 0.50 shows the point of equal preference; values above this line indicate greater preference while values below the line denote less preference. The bar colors match the food jar labels in Figure 1C. Individual rat preferences are shown as symbols in each bar. CF = chickpea flour; Y = low-fat vanilla yogurt; PB = creamy peanut butter; SFW = sugar–fat whip; ROD choices were high (H) or low (L) in fat (F) and/or sugar (S).

A separate set of naïve male rats was used to compare preferences between nutritionally similar food pairs from the ROD and HUM diets. When paired side by side as the only choices, the preference for the LFLS ROD choice was 0.905 (g) or 0.902 (kcal) compared to CF. Compared to Y, the preference for the ROD LFHS choice was 0.762 (g) or 0.938 (kcal) (Figure 11). The HFLS ROD choice preference was 0.505 (g) or 0.475 (kcal) compared to PB, and the ROD HFHS choice preference was 0.560 (g) or 0.529 (kcal) compared to SFW. In other words, when tested head-to-head, the preference for the high-fat choices on the HUM and ROD diets was similar, but the rats preferred the low-fat choices from the ROD diet over the low-fat HUM choices.

## 4. Discussion

The use of *cafeteria* or *choice* diets in rodent research is intended to better replicate human feeding conditions where there are often multiple palatable foods available. Therefore, an important test of fitness for any cafeteria-style choice diet is that the subjects find the options palatable and respond in a manner characteristic of the behavior observed when multiple palatable foods are available. Here, we show that rats find the human-food-based choice diets and custom choice rodent diets used here preferable to powdered chow, a conclusion supported by our observation that rats increase energy intake and the rate of ingestion on the HUM and the ROD choice diets compared to PC alone. Additionally, chow intake dropped when either choice diet was offered. There are other responses that changed similarly relative to PC on both choice diets. For example, on both the ROD and HUM diets, rats consumed more fat and sugar and less protein and nonsugar carbohydrate compared to PC alone. These results suggest that both diet arrays have utility for experiments seeking to present a palatable multiple-choice diet.

Although both the HUM and ROD diets induced responses indicative of increased palatability compared to PC alone, they were not identical in the behavioral response they evoked. In males and females, the dramatic increase in energy intake on HUM Day 1 was driven by both larger and more frequent meals. On the ROD diet, elevated Day 1 energy intake was driven by combined trends toward both more numerous and caloric meals in males but only by meal size in females. The response to Day 1 is important because it represents the first opportunity to experience the diets over a single day–night cycle. In male rats, the number or size of meals from both the ROD and HUM diets returned to PC levels by Day 8, but in females, the increased meal size persisted on the ROD diet. These data suggest that, although, on average, meal size drives elevated intake and the meal number is more stable when palatable foods are initially introduced, elevations in meal size *and* meal number may contribute to excess intake. Mechanistically, this indicates that both meal-initiation and meal-termination processes may be affected by the introduction of a palatable choice diet, but the effects on meal termination appear to be more long lasting.

While energy-intake and meal-pattern results were generally as we expected, this was not true for every measure. Given the nutritional similarity between the foods in the diet arrays, we expected the relative macronutrient intake to be similar between the HUM and ROD diets. Instead, we found that, compared to the HUM diet, the rats consumed less FAT and more CHO on the ROD diet array. It is possible that something about the nature of the foods drove the behavioral differences that we observed. One difference between HUM and ROD was that the protein content was standardized at 20% of kcal in the ROD choices compared to a range of 13.7–21.7% of kcal in the HUM food choices, resulting in the relative energy taken from PRO to average 3–4% lower on HUM compared to ROD; the increased proportion of PRO kcal would necessitate a decrease in the proportion of CHO and/or FAT kcal consumed on the ROD diet. In fact, in males and females, the proportion of energy taken from fat decreased by 10–11% while on ROD, while the proportion of energy taken from CHO increased by 6–8%. In other words, rats chose to make up the protein difference entirely from the proportion of fat calories consumed. The rats further reduced fat intake in favor of greater CHO intake while on the ROD diet. On the surface, this difference appeared to stem from the response to the ROD choices that were low in sugar; when the relative energy intake was assessed by food choice, the rats consumed more LFLS (ROD) compared with CF (HUM) and less HFLS (ROD) compared with PB, making it appear that the HFLS ROD choice was not as palatable to the rats, as was peanut butter from the HUM array. However, our side-by-side comparison revealed that rats nearly equally preferred the HF choices from the HUM and ROD diets but strongly preferred the LF ROD choices over their HUM counterparts. In total, intake during the choice-diet monitoring and during the two-jar tests suggests that rats ate more of the LFLS option at the expense of the HFLS option while on the ROD diet. These results demonstrate that it is critical to interpret *preference* in the context of all available food options.

It is not clear why, while presented with the ROD array, the rats chose to reduce intake of the HFLS choice in favor of eating more of the LFLS option. One possible factor is that all the ROD choices derived their fat from 10% soybean oil and 90% lard. It is therefore tempting to consider the possibility that a fatty-acid-specific mechanism made the ROD HF choices more similar to the rats, resulting in a relative decrease in variety and reducing the combined intake of the HF options, even though both were equally preferred to their HUM counterparts. The idea of a fatty-acid-specific mechanism would also provide a logical explanation for the greater fat intake observed when rats were presented with the HUM diet, because the fat in the HUM HF choices comes from differing sources (i.e., peanut butter, Crisco, or corn oil), ultimately increasing variety. Another possibility, or perhaps a contributing factor, is that cornstarch provides the nonsugar CHO in the ROD LFLS choice, and cornstarch is palatable to rats, for example, see references [26,27]. However, in Glass et al. [28], the rats chose a corn oil and shortening-based diet over a cornstarch-based diet, suggesting that fats are more preferred than cornstarch. Still, others have shown that cornstarch gels are preferred to powders by rats [26], indicating the form of the starch could be an important factor in preference. Although the ROD LFLS food and HUM chickpea flour were very similar in observable texture and nutritional content, rats preferred the ROD LFLS choice to CF when they were offered as the only two choices. It is possible that the relatively lower amylopectin content reduced the digestibility of the starch in CF [29,30], perhaps limiting the release of glucose monomers from the starch and reducing postingestive reinforcement for this choice.

Remarkably, although there were differences between the ROD and the HUM diets in the proportion of energy consumed from FAT and CHO, while on each diet, even when total kcal consumed declined significantly between Day 1 and Day 8, the proportion of energy from each macronutrient remained largely similar across the diet phase. The consistency of macronutrient intake across days suggests macronutrient intake is determined by the available foods and is strongly regulated.

Some behaviors we measured were only transiently affected by the introduction of palatable diets. For example, we found that energy intake, although altered on Day 1 of ROD or HUM access, approached PC levels by the end of the choice-diet phase. It is possible that, initially, the hedonic properties of the palatable choices overcame normal regulation of intake. Then, after the choices were available ad libitum for a period of days, their hedonic value decreased, thus changing the balance of the interaction between pathways driven by hedonics and those driven by physiological need. The decline in eating rate over time on the HUM diet supports the idea that the hedonic value of the diet decreased with continued exposure. Indeed, reduced motivation after repeated food exposure has been reported in humans and rodents, for example, [31,32]. Another possibility, not mutually exclusive to others, is that, after a period of elevated energy intake, long-term energy-balance signals (e.g., leptin) are potentiated, increasing the effectiveness of meal-termination signals, discussion of this idea can be found in references [33,34], and more effectively competing with hedonic drive. On the other hand, the eating rate on the ROD diet was stable over time in males and females, suggesting another factor could be involved. We cannot ignore the likelihood that the different food choices can alter the eating rate just by their physical nature, as the texture of the foods varies from powdery to pasty. Additionally, the transient changes of some behaviors that could indicate palatability, motivation, or hedonic value would suggest that the relatability of the model to the human-food environment could be increased by changing the food choices over the course of the experiment. Of course, this would increase the complexity of data analysis, and the utility of the approach would need to be considered in the context of the research question.

Although our study reveals some fundamental features of the responses of male and female rats to two different palatable cafeteria-style diets, it is not without limitations. In this study, the differences between the food choices in each diet array were focused on fat and sugar content and thus did not provide information about how manipulation of other nutrients or food characteristics might affect food choices and meal patterns. Rats were given each choice diet for 8 days and, although this is a relatively long time in the context of rodent food preference studies, it is possible that longer exposure to each diet could result in different findings. Finally, while five choices are very many relative to much of the food-choice research conducted in rodents, it still falls short of the nearly limitless options available to humans.

## 5. Conclusions

The primary objective of this study was to compare the responses of male and female rats to two different, but nutritionally similar, food arrays to assess their fitness for use as palatable cafeteria diets. Our findings show that, although both diet arrays induce responses suggesting utility, it appears that macronutrient composition is only part of what drives the choices of rats in multifood diet settings. Interpretative consideration must be given to the nature of food choices in the context of available options when interpreting cafeteria-diet intake. Additional research is needed to determine the mechanism driving differences in fat and carbohydrate intake between our two nutritionally similar diet arrays.

## Figures and Tables

**Table 1:** Characteristics of human-foods-based (HUM) and custom rodent (ROD) choice diets.

HUM Choices	%CHO	%SUG	%PRO	%FAT	kcal/g	ROD Choices	Research Diets No.	%CHO	%SUG	%PRO	%FAT	kcal/g
**PC**	57.9	9.0	28.7	13.4	3.36	**PC**	n/a	57.9	9.0	28.7	13.4	3.36
**CF**	71.5	3.4	17.0	11.5	3.92	**LFLS**	D21102802	67.0	4.0	20.0	13.0	3.79
**Y**	64.6	61.5	21.5	13.8	0.76	**LFHS**	D21102803	67.0	66.5	20.0	13.0	3.79
**PB**	15.7	5.9	13.7	70.6	6.18	**HFLS**	D21102804	10.0	4.0	20.0	70.0	5.41
**SFW**	28.6	27.5	13.6	57.8	5.76	**HFHS**	D21102801	25.4	24.8	20.0	54.6	4.85

Nutritionally similar foods between the human-foods choice diet (HUM) and custom rodent choice diet (ROD) are found in the same row. Row colors match the jar labels in Figure 1C and the abbreviation for each choice is shown in bold. Powdered rodent chow (PC) was an option on HUM and ROD. The other four choices on HUM were chickpea flour (CF), low-fat vanilla yogurt (Y), creamy peanut butter (PB), and sugar-fat whip (SFW). The choices on ROD are designated by their sugar (S) and fat (F) content being either high (H) or low (L) (i.e., LFLS = low-fat low-sugar, LFHS = low-fat high-sugar, HFLS = high-fat low-sugar, HFHS = high-fat, high-sugar). CHO = carbohydrate; SUG = sugar; PRO = protein.

**Table 2:** Energy and water intake statistics.

		Males		Females	
		Friedman Statistic	Bonferroni-Corrected *p*-Value	Friedman Statistic	Bonferroni-Corrected *p*-Value
**Energy Intake**	PC avg v HUM avg	**10.6066**	**<0.0001**	**5.3135**	**0.0003**
	PC avg v ROD avg	**6.3640**	**<0.0001**	**6.2796**	**<0.0001**
	HUM avg v ROD avg	**4.2426**	**0.0024**	0.9661	1.0512
	PC avg v HUM1	**11.0258**	**<0.0001**	**9.7900**	**<0.0001**
	PC avg v HUM8	**5.2832**	**<0.0001**	**3.5411**	**0.0030**
	HUM1 v HUM8	**5.7426**	**<0.0001**	**6.2490**	**<0.0001**
	PC avg v ROD1	**7.8099**	**<0.0001**	**7.4988**	**<0.0001**
	PC avg v ROD8	**4.5941**	**<0.0001**	**4.3743**	**0.0003**
	ROD1 v ROD8	**3.2159**	**0.0075**	**3.1245**	**0.0096**
	HUM1 v ROD1	**3.2159**	**0.0075**	3.0000	0.0597
**Relative** **Energy Intake**	PC avg v HUM avg	**3.1180**	**0.0228**	**5.6125**	**0.0003**
	PC avg v ROD avg	2.4944	0.0771	**5.6125**	**0.0003**
	HUM avg v ROD avg	0.6236	1.6287	0.0000	3.0000
	PC avg v HUM1	**6.2796**	**<0.0001**	**7.5609**	**<0.0001**
	PC avg v HUM8	0.9661	1.0512	2.1602	0.1458
	HUM1 v HUM8	**5.3135**	**0.0003**	**5.4006**	**0.0003**
	PC avg v ROD1	**5.6125**	**0.0003**	**6.7626**	**<0.0001**
	PC avg v ROD8	0.0000	3.0000	**3.3813**	**0.0135**
	ROD1 v ROD8	**5.6125**	**0.0003**	**3.3813**	**0.0135**
	HUM1 v ROD1	0.0000	3.0000	2.1413	0.1143
**Water Intake**	PC avg v HUM avg	**13.5607**	**<0.0001**	**13.2400**	**<0.0001**
	PC avg v ROD avg	**6.4037**	**<0.0001**	**5.0097**	**<0.0001**
	HUM avg v ROD avg	**7.1570**	**<0.0001**	**8.2303**	**<0.0001**
	PC avg v HUM1	**10.6066**	**<0.0001**	**10.6066**	**<0.0001**
	PC avg v HUM8	**6.3640**	**<0.0001**	**6.3640**	**<0.0001**
	HUM1 v HUM8	**4.2426**	**0.0024**	**4.2426**	**0.0024**
	PC avg v ROD1	**10.1705**	**<0.0001**	**10.6066**	**<0.0001**
	PC avg v ROD8	**3.0135**	**0.0132**	**6.3640**	**<0.0001**
	ROD1 v ROD8	**7.1570**	**<0.0001**	**4.2426**	**<0.0001**
	HUM1 v ROD1	**7.5337**	**<0.0001**	**8.9460**	**<0.0001**

Within each sex, the averages for each measure on each diet were compared, the first and last day of each diet were compared to the PC average and to each other, and Day 1 on the choice diets were compared. PC avg = mean value from the PC days preceding each choice-diet phase; HUM avg = mean during the HUM diet; HUM1 = HUM diet Day 1; HUM8 = HUM diet Day 8; ROD avg = mean during the ROD diet; ROD1 = ROD diet Day 1; ROD8 = ROD diet Day 8. Statistics with *p*-value ≤ 0.05 are bolded.

**Table 3:** Energy and water intake statistics for males vs. females.

		U	*p*-Value
**Energy Intake**	**PC avg**	**0.0000**	**0.0056**
	**HUM avg**	**0.0000**	**0.0056**
	**HUM Day 1**	**0.0000**	**0.0056**
	**HUM Day 8**	**1.0000**	**0.0077**
	**ROD avg**	**2.0000**	**0.0112**
	**ROD Day 1**	**2.0000**	**0.0112**
	**ROD Day 8**	**0.0000**	**0.0056**
**Relative** **Energy Intake**	PC avg	52.0000	0.2499
	HUM avg	52.0000	0.2499
	HUM Day 1	44.0000	1.0000
	HUM Day 8	51.0000	0.3220
	ROD avg	54.0000	0.1463
	ROD Day 1	40.0000	1.0000
	ROD Day 8	56.0000	0.0819
**Water Intake**	**PC avg**	**0.0000**	**0.0056**
	HUM avg	12.0000	0.2499
	HUM Day 1	32.0000	1.0000
	HUM Day 8	16.0000	0.6503
	**ROD avg**	**0.0000**	**0.0056**
	ROD Day 1	11.5000	0.2184
	**ROD Day 8**	**0.0000**	**0.0056**

Statistics with *p*-value ≤ 0.05 are bolded.

**Table 4:** Relative food intake statistics.

		Males		Females	
		Friedman Statistic	Bonferroni-Corrected *p*-Value	Friedman Statistic	Bonferroni-Corrected *p*-Value
**PC**	HUM avg v ROD avg	1.5954	0.3588	1.1741	0.7449
	HUM1 v HUM8	**4.9858**	**<0.0001**	0.3914	2.0937
	ROD1 v ROD8	**3.3903**	**0.0051**	1.1741	0.7449
	HUM1 v ROD1	0.3989	2.0772	0.9784	1.0038
**CF/** **LFLS**	HUM avg v ROD avg	**5.2647**	**<0.0001**	**3.5355**	**0.0036**
	HUM1 v HUM8	1.1445	0.7806	1.7678	0.2574
	ROD1 v ROD8	0.4578	1.9497	1.0607	0.8883
	HUM1 v ROD1	**5.4936**	**<0.0001**	**2.6517**	**0.0360**
**Y/** **LFHS**	HUM avg v ROD avg	0.6709	1.5201	0.2667	2.3739
	HUM1 v HUM8	0.9393	1.0620	0.2667	2.3739
	ROD1 v ROD8	1.6102	0.3489	2.1337	0.1197
	HUM1 v ROD1	0.2684	2.3700	1.2002	0.7143
**PB/** **HFLS**	HUM avg v ROD avg	**6.4550**	**<0.0001**	**2.9580**	**0.0165**
	HUM1 v HUM8	**4.8412**	**<0.0001**	0.0000	3.0000
	ROD1 v ROD8	**3.5502**	**0.0033**	**3.8032**	**0.0015**
	HUM1 v ROD1	**8.3915**	**<0.0001**	**2.5355**	**0.0477**
**SFW/** **HFHS**	HUM avg v ROD avg	0.2643	2.3793	0.9917	0.9846
	HUM1 v HUM8	0.2643	2.3793	0.1417	2.6646
	ROD1 v ROD8	0.2643	2.3793	**2.5500**	**0.0459**
	HUM1 v ROD1	0.2643	2.3793	1.2750	0.6321

Within each sex, the averages for each measure on each diet were compared, the first and last day of each diet were compared to each other, and Day 1 on the choice diets were compared. PC avg = mean value from the PC days preceding each choice-diet phase; HUM avg = mean during the HUM diet; HUM1 = HUM diet Day 1; HUM8 = HUM diet Day 8; ROD avg = mean during the ROD diet; ROD1 = ROD diet Day 1; ROD8 = ROD diet Day 8. Statistics with *p*-value ≤ 0.05 are bolded.

**Table 5:** Relative food intake statistics for males vs. females.

		U	*p*-Value
**PC ** **Intake**	**HUM avg**	**0.0000**	**0.0024**
	HUM Day 1	16.0000	0.2181
	**HUM Day 8**	**0.0000**	**0.0018**
	ROD avg	12.0000	0.1071
	ROD Day 1	33.0000	1.0000
	ROD Day 8	12.0000	0.0930
**CF/LFLS** **Intake**	HUM avg	42.0000	0.8808
	HUM Day 1	52.0000	0.1071
	HUM Day 8	24.0000	1.0000
	ROD avg	22.0000	0.8808
	ROD Day 1	37.0000	1.0000
	ROD Day 8	22.000	0.8808
**Y/LFHS** **Intake**	HUM avg	35.0000	1.0000
	HUM Day 1	24.0000	1.0000
	HUM Day 8	37.0000	1.0000
	ROD avg	40.0000	1.0000
	ROD Day 1	36.0000	1.0000
	ROD Day 8	34.0000	1.0000
**PB/HFLS** **Intake**	HUM avg	24.0000	1.0000
	HUM Day 1	13.0000	0.1380
	HUM Day 8	42.0000	0.8808
	ROD avg	45.0000	0.5166
	ROD Day 1	45.0000	0.5166
	ROD Day 8	33.0000	1.0000
**SFW/HFHS** **Intake**	HUM avg	38.0000	1.0000
	HUM Day 1	37.0000	1.0000
	HUM Day 8	37.0000	1.0000
	ROD avg	38.0000	1.0000
	ROD Day 1	27.0000	1.0000
	ROD Day 8	39.0000	1.0000

Statistics with *p*-value ≤ 0.05 are bolded.

**Table 6:** Relative macronutrient intake statistics.

		Males		Females	
		Friedman Statistic	Bonferroni-Corrected *p*-Value	Friedman Statistic	Bonferroni-Corrected *p*-Value
**CHO** **Intake**	PC avg v HUM avg	**6.7626**	**<0.0001**	**10.6066**	**<0.0001**
	PC avg v ROD avg	**3.3813**	**0.0135**	**6.3640**	**<0.0001**
	HUM avg v ROD avg	**3.3813**	**0.0135**	**4.2426**	**0.0024**
	PC avg v HUM1	**7.5609**	**<0.0001**	**5.6125**	**0.0003**
	PC avg v HUM8	**5.4006**	**0.0003**	**5.6125**	**0.0003**
	HUM1 v HUM8	2.1602	0.1458	0.0000	3.0000
	PC avg v ROD1	**3.1180**	**0.0228**	**5.4006**	**0.0003**
	PC avg v ROD8	2.4944	0.0771	**7.5609**	**<0.0001**
	ROD1 v ROD8	0.6236	1.6287	2.1602	0.1458
	HUM1 v ROD1	1.5275	0.5115	1.5275	0.5115
**SUGAR** **Intake**	PC avg v HUM avg	**7.5609**	**<0.0001**	**5.6125**	**0.0003**
	PC avg v ROD avg	**5.4006**	**0.0003**	**5.6125**	**0.0003**
	HUM avg v ROD avg	2.1602	0.1458	0.0000	3.0000
	PC avg v HUM1	**5.6125**	**0.0003**	**5.6125**	**0.0003**
	PC avg v HUM8	**5.6125**	**0.0003**	**5.6125**	**0.0003**
	HUM1 v HUM8	0.0000	3.0000	0.0000	3.0000
	PC avg v ROD1	**10.6066**	**<0.0001**	**6.2796**	**<0.0001**
	PC avg v ROD8	**6.3640**	**<0.0001**	**6.3135**	**0.0003**
	ROD1 v ROD8	**4.2426**	**0.0024**	0.9661	1.0512
	HUM1 v ROD1	0.6831	1.5495	0.0000	3.0000
**FAT** **Intake**	PC avg v HUM avg	**10.6066**	**<0.0001**	**7.3196**	**<0.0001**
	PC avg v ROD avg	**6.3640**	**<0.0001**	**3.0819**	**0.0108**
	HUM avg v ROD avg	**4.2426**	**0.0024**	**4.2376**	**0.0003**
	PC avg v HUM1	**7.5609**	**<0.0001**	**5.6125**	**0.0003**
	PC avg v HUM8	**5.4006**	**0.0003**	**5.6125**	**0.0003**
	HUM1 v HUM8	2.1602	0.1458	0.0000	3.0000
	PC avg v ROD1	**6.2796**	**<0.0001**	**5.4006**	**0.0003**
	PC avg v ROD8	**5.3135**	**0.0003**	**7.5609**	**<0.0001**
	ROD1 v ROD8	0.9661	1.0512	2.1602	0.1458
	HUM1 v ROD1	3.0000	0.0597	**4.0450**	**0.0006**
**PRO** **Intake**	PC avg v HUM avg	**14.1421**	**<0.0001**	**11.3837**	**<0.0001**
	PC avg v ROD avg	**4.5962**	**<0.0001**	**3.8944**	**0.0009**
	HUM avg v ROD avg	**9.5459**	**<0.0001**	**7.4893**	**<0.0001**
	PC avg v HUM1	**10.6066**	**<0.0001**	**5.6125**	**0.0003**
	PC avg v HUM8	**6.3640**	**<0.0001**	**5.6125**	**0.0003**
	HUM1 v HUM8	**4.2426**	**0.0024**	0.0000	3.0000
	PC avg v ROD1	**6.2796**	**<0.0001**	**5.3135**	**0.0003**
	PC avg v ROD8	**5.3135**	**0.0003**	**6.2796**	**<0.0001**
	ROD1 v ROD8	0.9661	1.0512	0.9661	1.0512
	HUM1 v ROD1	**8.4853**	**<0.0001**	**6.5906**	**<0.0001**

Within each sex, the averages for each measure on each diet were compared, the first and last day of each diet were compared to the PC average and to each other, and Day 1 on the choice diets were compared. CHO = carbohydrate; PC avg = mean value from the PC days preceding each choice-diet phase; HUM avg = mean during the HUM diet; HUM1 = HUM diet Day 1; HUM8 = HUM diet Day 8; ROD avg = mean during the ROD diet; ROD1 = ROD diet Day 1; ROD8 = ROD diet Day 8. Statistics with *p*-value ≤ 0.05 are bolded.

**Table 7:** Relative macronutrient intake statistics for males vs. females.

		U	*p*-Value
**CHO ** **Intake**	HUM avg	32.0000	1.0000
	HUM Day 1	42.0000	1.0000
	HUM Day 8	23.0000	1.0000
	ROD avg	26.0000	1.0000
	ROD Day 1	32.0000	1.0000
	ROD Day 8	25.0000	1.0000
**SUG ** **Intake**	HUM avg	41.0000	1.0000
	HUM Day 1	23.0000	1.0000
	HUM Day 8	38.0000	1.0000
	ROD avg	43.0000	1.0000
	ROD Day 1	35.0000	1.0000
	ROD Day 8	40.0000	1.0000
**FAT ** **Intake**	HUM avg	34.0000	1.0000
	HUM Day 1	24.0000	1.0000
	HUM Day 8	42.0000	1.0000
	ROD avg	41.0000	1.0000
	ROD Day 1	32.0000	1.0000
	ROD Day 8	41.0000	1.0000
**PRO ** **Intake**	HUM avg	14.0000	0.3522
	HUM Day 1	28.0000	1.0000
	HUM Day 8	16.0000	0.5574
	ROD avg	10.0000	0.1254
	ROD Day 1	35.0000	1.0000
	ROD Day 8	12.0000	0.2112

**Table 8:** Meal number and meal size statistics.

		Males		Females	
		Friedman Statistic	Bonferroni-Corrected *p*-Value	Friedman Statistic	Bonferroni-Corrected *p*-Value
**Number of Meals**	PC avg v HUM avg	0.2357	2.4513	0.7568	1.3851
	PC avg v ROD avg	0.2357	2.4513	0.0000	3.0000
	HUM avg v ROD avg	0.4714	1.9338	0.7568	1.3851
	PC avg v HUM1	**3.2051**	**0.0192**	**3.1180**	**0.0228**
	PC avg v HUM8	0.3374	2.2224	0.6236	1.6287
	HUM1 v HUM8	**2.8677**	**0.0372**	2.4944	0.0771
	PC avg v ROD1	2.2215	0.1299	0.1231	2.7114
	PC avg v ROD8	0.1587	2.6286	0.1231	2.7114
	ROD1 v ROD8	2.0629	0.1746	0.2462	2.4273
	HUM1 v ROD1	2.3760	0.1476	3.0000	0.0597
**Meal Size** **(kcal)**	PC avg v HUM avg	**5.3072**	**0.0003**	**5.3135**	**0.0003**
	PC avg v ROD avg	2.0412	0.1815	**6.2796**	**<0.0001**
	HUM avg v ROD avg	**3.2660**	**0.0168**	0.9661	1.0512
	PC avg v HUM1	**3.5824**	**0.0090**	**3.5824**	**0.0090**
	PC avg v HUM8	2.2797	0.1164	1.3027	0.6411
	HUM1 v HUM8	1.3027	0.6411	2.2797	0.1164
	PC avg v ROD1	2.5677	0.0669	**4.4900**	**0.0015**
	PC avg v ROD8	1.7118	0.3270	**3.3675**	**0.0138**
	ROD1 v ROD8	0.8559	1.2195	1.1225	0.8415
	HUM1 v ROD1	3.0000	0.0597	0.0000	3.0000

Within each sex, the averages for each measure on each diet were compared, the first and last day of each diet were compared to the PC average and to each other, and Day 1 on the choice diets were compared. PC avg = mean value from the PC days preceding each choice-diet phase; HUM avg = mean during the HUM diet; HUM1 = HUM diet Day 1; HUM8 = HUM diet Day 8; ROD avg = mean during the ROD diet; ROD1 = ROD diet Day 1; ROD8 = ROD diet Day 8. Statistics with *p*-value ≤ 0.05 are bolded.

**Table 9:** Meal-pattern statistics for males vs. females.

		U	*p*-Value
**Number of Meals**	PC avg	31.0000	1.0000
	HUM avg	31.5000	1.0000
	HUM Day 1	33.0000	1.0000
	HUM Day 8	32.0000	1.0000
	ROD avg	27.5000	1.0000
	ROD Day 1	20.0000	1.0000
	ROD Day 8	22.5000	1.0000
**Meal Size (kcal)**	**PC avg**	**4.0000**	**0.0231**
	**HUM avg**	**5.0000**	**0.0322**
	HUM Day 1	9.0000	0.1099
	HUM Day 8	9.0000	0.0889
	ROD avg	10.0000	0.1463
	ROD Day 1	15.0000	0.5194
	ROD Day 8	18.0000	0.9905
**Meal Duration (min)**	PC avg	17.0000	0.8064
	HUM avg	23.0000	1.0000
	HUM Day 1	35.0000	1.0000
	HUM Day 8	18.0000	0.9905
	ROD avg	14.0000	0.4109
	ROD Day 1	18.0000	0.9905
	ROD Day 8	25.0000	1.0000
**Meal Eating Rate (kcal/min)**	PC avg	10.0000	0.1463
	HUM avg	24.0000	1.0000
	HUM Day 1	13.0000	0.3220
	HUM Day 8	24.0000	1.0000
	ROD avg	27.0000	1.0000
	ROD Day 1	31.0000	1.0000
	ROD Day 8	30.0000	1.0000
**Foods/Meal**	PC avg	13.0000	0.3220
	HUM avg	11.0000	0.1918
	HUM Day 1	22.0000	1.0000
	HUM Day 8	13.0000	0.3220
	ROD avg	8.0000	0.0819
	ROD Day 1	17.0000	0.8064
	ROD Day 8	18.0000	0.9821

Statistics with *p*-value ≤ 0.05 are bolded.

**Table 10:** Meal duration, rate, and foods/meals statistics.

		Males		Females	
		Friedman Statistic	Bonferroni-Corrected *p*-Value	Friedman Statistic	Bonferroni-Corrected *p*-Value
**Meal Duration ** **(min)**	PC avg v HUM avg	1.9522	0.2136	2.4944	0.0771
	PC avg v ROD avg	2.2311	0.1275	**3.1180**	**0.0228**
	HUM avg v ROD avg	0.2789	2.3532	0.6236	1.6287
	PC avg v HUM1	**5.3072**	**0.0003**	2.5677	0.0669
	PC avg v HUM8	2.0412	0.1815	1.7118	0.3270
	HUM1 v HUM8	**3.2660**	**0.0168**	0.8559	1.2195
	PC avg v ROD1	2.2311	0.1275	**2.7681**	**0.0453**
	PC avg v ROD8	1.9522	0.2136	**2.7681**	**0.0453**
	ROD1 v ROD8	0.2789	2.3532	0.0000	3.0000
	HUM1 v ROD1	3.0000	0.0597	0.6831	1.5495
**Meal Eating Rate (kcal/min)**	PC avg v HUM avg	**5.6125**	**0.0003**	**3.3675**	**0.0138**
	PC avg v ROD avg	**5.6125**	**0.0003**	**4.4900**	**0.0015**
	HUM avg v ROD avg	0.0000	3.0000	1.1225	0.8415
	PC avg v HUM1	**7.8619**	**<0.0001**	**5.3072**	**0.0003**
	PC avg v HUM8	**3.1448**	**0.0090**	2.0412	0.1815
	HUM1 v HUM8	**4.7171**	**<0.0001**	**3.2660**	**0.0168**
	PC avg v ROD1	**5.6125**	**0.0003**	**2.7681**	**0.0453**
	PC avg v ROD8	**5.6125**	**0.0003**	**2.7681**	**0.0453**
	ROD1 v ROD8	0.0000	3.0000	0.0000	3.0000
	HUM1 v ROD1	3.0000	0.0597	1.5275	0.5115
**Foods/Meal**	PC avg v HUM avg	**3.3813**	**0.0135**	**5.4006**	**0.0003**
	PC avg v ROD avg	**6.7626**	**<0.0001**	**7.5609**	**<0.0001**
	HUM avg v ROD avg	**3.3813**	**0.0135**	2.1602	0.1458
	PC avg v HUM1	**3.5824**	**0.0090**	**3.9605**	**0.0042**
	PC avg v HUM8	1.3027	0.6411	**3.6004**	**0.0087**
	HUM1 v HUM8	2.2797	0.1164	0.3600	2.1726
	PC avg v ROD1	**3.1180**	**0.0228**	2.5677	0.0669
	PC avg v ROD8	2.4944	0.0771	1.7118	0.3270
	ROD1 v ROD8	0.6236	1.6287	0.8559	1.2195
	HUM1 v ROD1	0.6831	1.5495	0.6831	1.5495

Within each sex, the averages for each measure on each diet were compared, the first and last day of each diet were compared to the PC average and to each other, and Day 1 on the choice diets were compared. PC avg = mean value from the PC days preceding each choice-diet phase; HUM avg = mean during the HUM diet; HUM1 = HUM diet Day 1; HUM8 = HUM diet Day 8; ROD avg = mean during the ROD diet; ROD1 = ROD diet Day 1; ROD8 = ROD diet Day 8. Statistics with *p*-value ≤ 0.05 are bolded.

## Data Availability

The data presented in this study are available on request from the corresponding author.
